# How to engage communities on a large scale? Lessons from World Mosquito Program in Rio de Janeiro, Brazil

**DOI:** 10.12688/gatesopenres.13153.2

**Published:** 2021-02-04

**Authors:** Guilherme B. Costa, Ruth Smithyman, Scott L. O'Neill, Luciano A. Moreira

**Affiliations:** 1 World Mosquito Program Brasil, Fundação Oswaldo Cruz - FIOCRUZ, Rio de Janeiro, Rio de Janeiro, 20930-040, Brazil; 2 Institute of Vector-Borne Disease, Monash University, Clayton, Victoria, 3800, Australia; 3 Mosquitos Vetores: Endossimbiontes e Interação Patógeno-Vetor, Instituto René Rachou - Fiocruz, Belo Horizonte, Minas Gerais, 30190-002, Brazil

**Keywords:** Wolbachia, Community Engagement, Vector Control, Aedes aegypti

## Abstract

One of the pillars of the World Health Organization’s (WHO) Global Vector Control Response 2017–2030 strategy is the engagement of communities. Among the priority activities, defined by 2022 by the WHO, is the development of plans for the effective engagement and mobilisation of communities in vector control. Novel technologies for arboviruses control are being developed, such as the *Wolbachia* method, implemented by the World Mosquito Program (WMP). Here we discuss and analyse the framework for community engagement implemented by the WMP in Brazil, during the large-scale deployment of the method in the municipalities of Niterói and Rio de Janeiro, Brazil. Our experience indicates that the community engagement work for arboviruses control should be understood as an opportunity for local development. It is necessary, based on an integrated analysis of the territory, to understand that the actions for arboviruses control could be a catalyst for the necessary socioenvironmental, cultural and public health changes. Furthermore, it is essential to understand that community engagement goes beyond informing or asking for population consent, but it constitutes a possibility for dialogue and exchange between the various stakeholders present in the territories, to build on cooperation for mosquito-borne disease control.

## Introduction

Arboviruses, or Arthropod-borne viruses, such as dengue, Zika and chikungunya, have quickly spread to new territories. Diseases previously restricted to countries in tropical and subtropical areas are already beginning to appear in temperate zones. Unplanned urban development, land-use changes, climate change and increased international mobility are some possible explanations for their dissemination. The World Health Organization (WHO) estimates that 700 thousand deaths from vector-borne diseases occur annually, and currently, 80% of the world population live in areas at risk from at least one major vector-borne disease ^1^.

To address this challenge and reduce threats, the WHO has defined an action strategy for member countries to be implemented by 2030. The Global Vector Control Response 2017–2030 calls for “improved public health entomology (and malacology) capacity and capability, a well-defined national research agenda, better coordination within and between sectors, community involvement in vector control, strengthened monitoring systems and novel interventions with proven effectiveness ^1^”.

One of the pillars of the WHO strategy is the engagement and mobilisation of communities, and among the priority activities until 2022 is the development of national plans for the effective engagement and mobilisation of communities in vector control. The WHO recommendations are supported by the scientific literature, which indicates that the approach and engagement with communities directly impacted by arboviruses make control actions feasible and can positively impact on their success ^2– 11^.

WHO also reminds us that communities have a key role not only in successfully controlling arboviruses, but for their sustainability, and that engagement actions must be systematised and shared: “documentation of existing community engagement strategies and their impact should be undertaken in order to share relevant best practices within and between countries ^1^”.

This paper fits into this context and presents the framework for community engagement given by the World Mosquito Program in Brazil (WMP Brazil), a not-for-profit initiative that works to protect the global community from mosquito-borne diseases by introducing the wMel strain of *Wolbachia* into *Aedes aegypti* mosquitoes.

## The role of community engagement for arboviruses control

Whatever the action to control arboviruses, at some point, it will involve the participation of the population. The traditional mechanical method requires community participation to eliminate breeding sites. In new technologies, as in the case of WMP Brazil, participation can take place in the release of *Ae. aegypti* with *Wolbachia*, hosting and monitoring mosquito traps or participating in local committees to monitor the progress of this initiative. In general, we understand that for the control of arboviruses the community must be involved, either for operational reason, collaborating in the prevention and control of these diseases, or ethical reason, holding the right to be informed and consent to the action to be taken.

From an operational point of view, community participation is essential for successful arbovirus control ^2^, and the main role of community engagement in this regard is to enable the mobilisation of individuals and social groups so that it is possible to achieve the desired results. An example is a study conducted by Sánchez *et al.* ^10^, in Cuba. For two years, the team carried out community actions in three areas of the municipality of Playa, Ciudade de la Habana, with the aim of increasing the participation of the population in the fight against the dengue mosquito. The actions progressed according to the context and local technical capacities, with different work approaches in each area. At the end of the period, the *Ae. aegypti* infestation rate decreased by 79% and there were no cases of dengue in the same areas.

**Figure 1.**
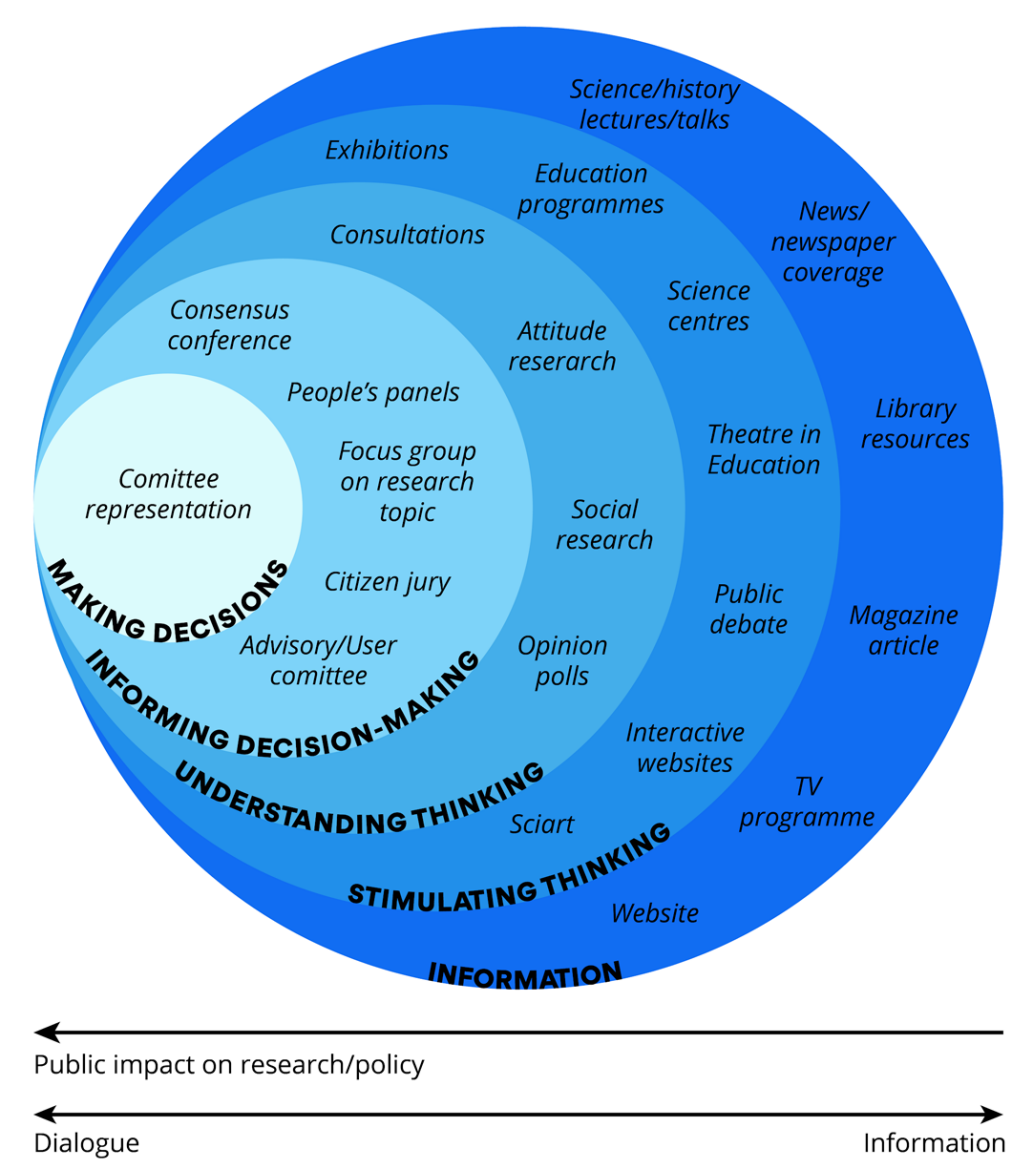
Welcome Trust's "onion"model (Wellcome Trust ^17^).

This case and others described in the literature ^2, 3, 5, 7, 10^ point out that in order to achieve effective community engagement, it is necessary to map and analyse the local sociopolitical and economic context in order to develop a strategy adjusted to that territory and to enable mobilisation. We understand that this analysis is fundamental considering that the participation of the citizen in the public space, although it is perceived in common sense as something naturally given, is, as described in the literature, an educational process to be developed ^12– 14^ and is directly impacted by the organisation of public space and local political culture ^15^. Therefore, it seems to us essential, when thinking about the population’s commitment to the control of arboviruses, that the following question is answered: what do we want from the participation of the population in the prevention or control of these diseases? From informational actions that seek the consent of the population, to the effective dialogue between scientists, health managers and the community with a view to developing cooperative actions, it is possible to undertake several engagement actions. In this sense, we share the understanding of the researchers gathered at the “Community Engagement - Under the Microscope” workshop, that community engagement can be organised like the layers of an onion ( Figure 1).

The proposed model, inspired by Arnstein’s ^16^ ladder of citizen participation, is particularly interesting when we think about large scale community engagement. Considering the local culture and political context, the greater the plurality of actions, the more effective the engagement will be, as it will allow each subject to be impacted and involved in a particular way. At the same time that it is necessary to ensure open channels of communication to inform and receive information from the community, it is necessary to enable community spaces for dialogue, where scientists and members of the community will exchange knowledge, after all, as Dunn ^17^ points out: “engagement is about ‘exchange’. It is not just about providing information or disseminating ideas or results. Engagement challenges the notion of communities as ‘recipients’ and has the potential for community members to become politically and critically aware of scientific processes” (Dunn, 2012:3).

This understanding leads us to the ethical perspective of community engagement, especially when it comes to the development of new technologies, as is the case with mosquitoes with *Wolbachia* from the World Mosquito Program. King *et al.* ^18^ remind us that the participation of the population enables the identification and management of non-obvious risks, the extension of respect for community stakeholders, seeing them as people and not just as facilitating participants in the action. In addition, the participation of the various groups and individuals that make up the community is not uniform or guided by a single interest. Each stakeholder takes collective action to their own interests and motivations and influences the public space of participation ^12, 15^. Identifying and managing these interests is also the role of community engagement, especially when the maximum expression of this is understood, still in reference to the model proposed by the Wellcome Trust ( Figure 1), as an action that enables dialogue between the parties. By dialogue, we understand that it is a way to concentrate the energy of the differences present in the territory to build something new that brings benefits for collectivity. In the dialogue, there are no sides, nor someone who has exclusive knowledge. Assuming Massardier’s ^19^ perspective that policymaking is to manage the collective actions of the actors, is to hold them together, no longer by authority, but by understanding the different rationality of each actor, we can understand that the dialogue fulfils a fundamental role in the management of a mosquito borne-disease policies.

King *et al.* ^18^ also indicate that from an ethical point of view, the participation of the population builds legitimacy to scientific action (and we understand that also to health management and arbovirus control actions):

“community engagement embodies a democratic ideal in which legitimacy emerges from deliberative processes through which disagreement is acknowledged and addressed, lines of accountability are established between the stakeholder community and researchers, and stakeholders are empowered to ask directly for justification regarding the trial’s conduct and management (King *et al.*, 2014, p.3)”.

And the authors complete that when identifying and considering the interests and concerns of diverse groups and individuals, creates “the human infrastructure necessary to support the deliberations and discussions that are necessary to discover, and to be responsive to, this range of interests (of the community)” (King *et al.*, 2014, p. 4). It is the role of community engagement, therefore, to respect and consider the different interests of the communities involved in combating arboviruses.

## The World Mosquito Program’s community engagement experience in Brazil

The World Mosquito Program (WMP), formerly Eliminate Dengue, is a global initiative to combat arboviruses. Through the introduction of the endosymbiotic bacteria *Wolbachia* into the *Aedes aegypti* population, the objective of the WMP is to reduce the transmission of dengue, Zika, chikungunya, yellow fever and Mayaro fever.

**Figure 2.**
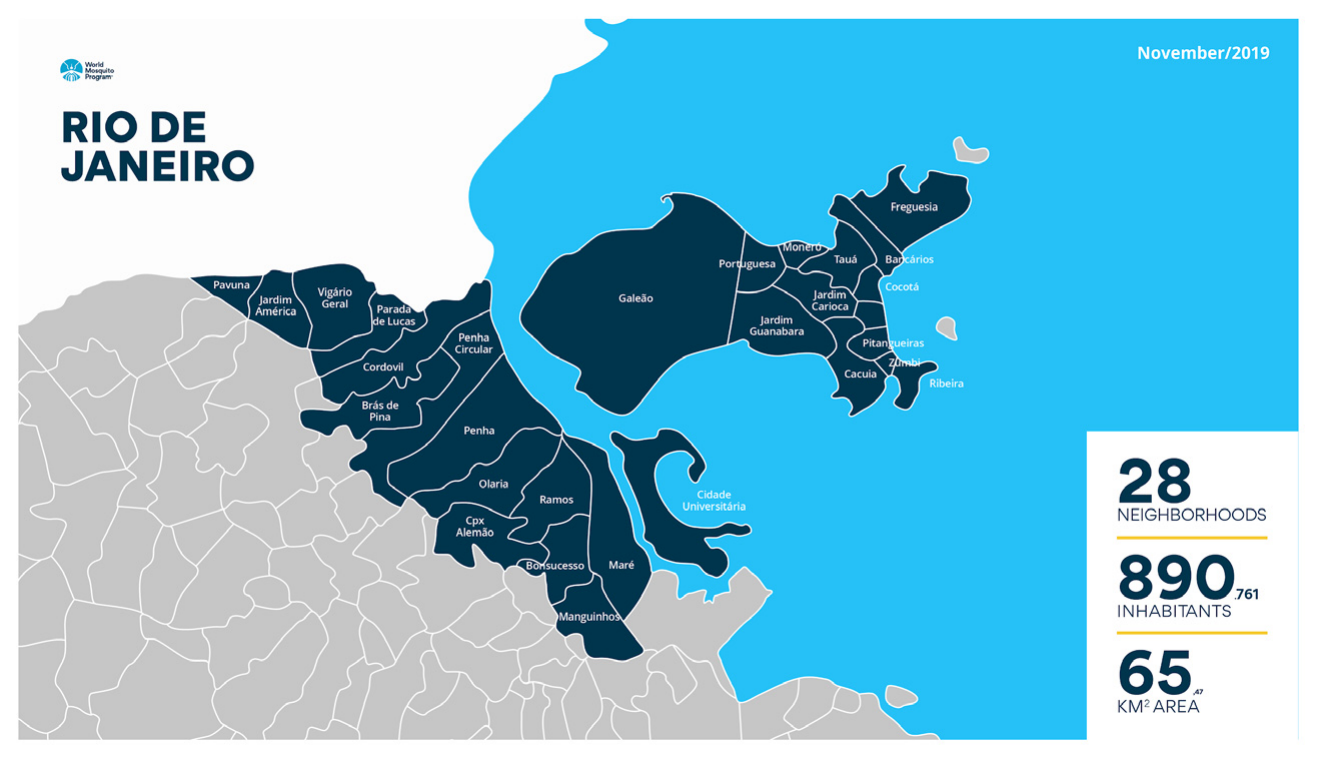
World Mosquito Program (WMP) Brazil's release area at Rio de Janeiro.

**Figure 3.**
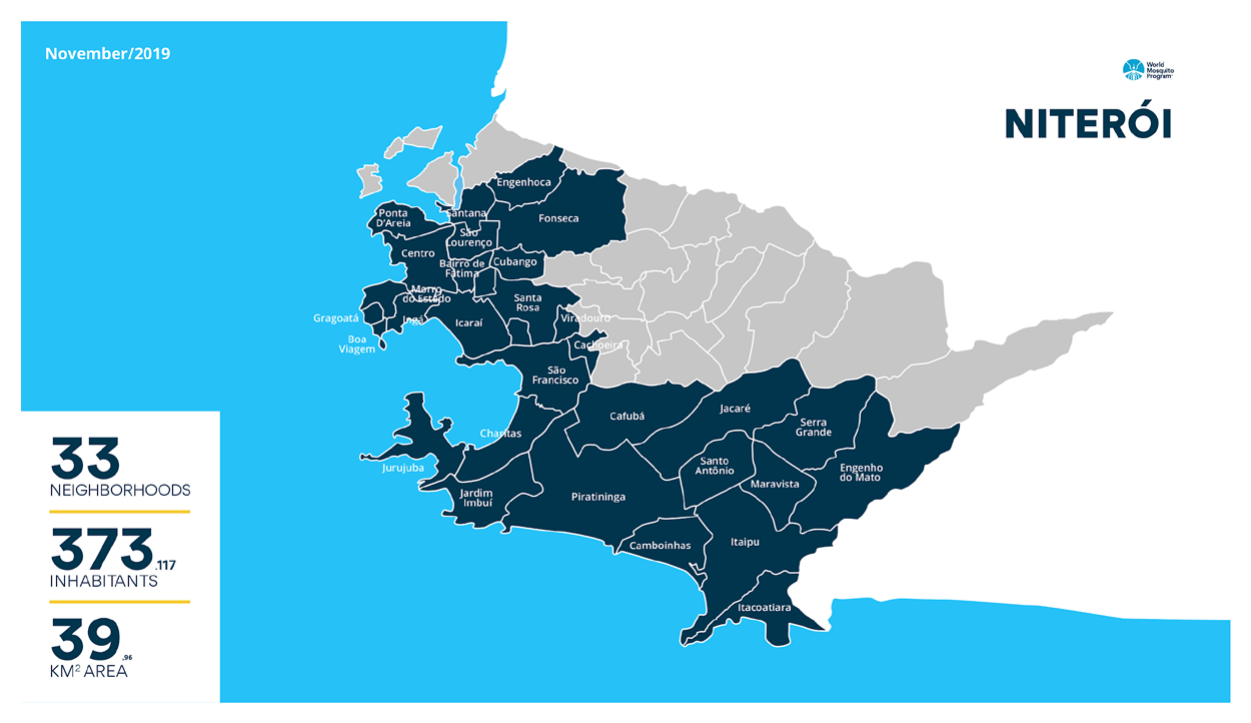
World Mosquito Program (WMP) Brazil's release area at Niterói.

Studies conducted by different groups ^20– 26^ indicate that *Ae. aegypti* that carry *Wolbachia* have a reduced ability to transmit these viruses to people, decreasing the risk of outbreaks of these arboviruses. Once mosquitoes with *Wolbachia* are released into the environment, they breed with field mosquitoes, and consequently, the prevalence of *Wolbachia*-positive mosquitoes increases and tends to become stable. Currently, the WMP operates in 12 countries, including Brazil, where releases of *Aedes aegypti* mosquitoes with *Wolbachia* have occurred since 2014 in the municipalities of Rio de Janeiro (RJ) and Niterói (RJ). After a pilot phase, the project has expanded its activities since 2016 and, by the end of 2019, it has reached an area of 105 km ^2^, which covers 1,263,878 inhabitants ( Figure 2 and Figure 3).

In order to operate in Brazil, the WMP obtained approval from national regulatory bodies, such as the National Health Surveillance Agency (Anvisa), the Brazilian Institute of the Environment and Renewable Natural Resources (Ibama) and the Ministry of Agriculture, Livestock and Food Supply (MAPA). It also obtained authorization from the National Research Ethics Commission (CONEP, in Portuguese) and maintains an advisory committee of specialists, bringing together researchers from different areas of knowledge, experts in arboviruses and community engagement.

**Figure 4.**
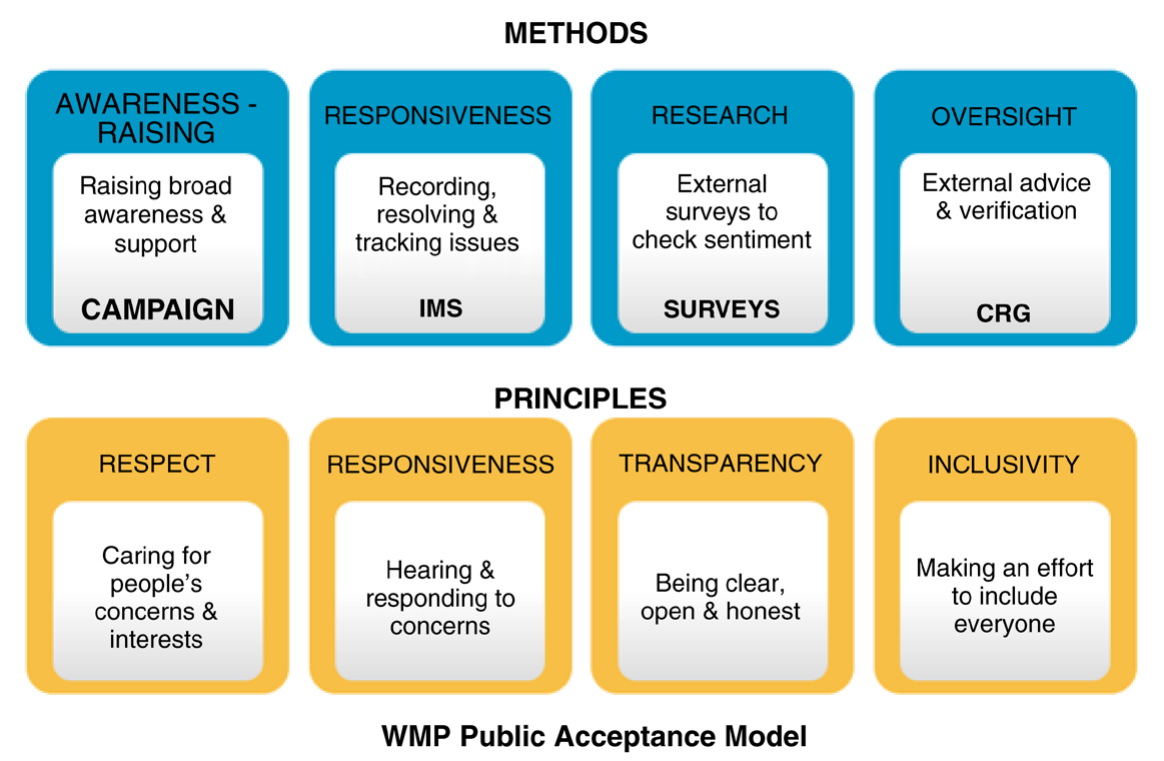
World Mosquito Program (WMP) Public Acceptance Model.

Before the release of mosquitoes with *Wolbachia*, WMP carried out communication and community engagement actions in the areas where it operates. To this end, it elaborated the Public Acceptance Model (PAM), which is currently the framework that guides the countries that participate in this initiative. PAM was designed to protect the rights of communities through rigorous processes and protective measures appropriate to the perceived risks of the intervention. These measures are underpinned by the principles of respect, transparency, inclusivity and responsiveness ^27– 29^. PAM has the following pillars: 1) the implementation of communication and engagement campaigns, whose objective is to generate knowledge and engagement of the population regarding the WMP *Wolbachia* Method; 2) the creation of a Community Reference Group, with the objective to create a dialogue relationship with the community through this consultative committee formed by representatives from different sectors in the areas in which it operates; 3) the implementation of an incident registration system, which has the role of registering and answering the population’s doubts and concerns regarding the method; and 4) conducting community surveys, the main objective of which is to measure the level of awareness and approval of the method by the community. Figure 4 illustrates the PAM.

In Brazil, communication and community engagement activities were conducted by a multidisciplinary team, composed of professionals in the areas of communication, biology, environmental science, geography, environmental engineering and social work. Altogether, 13 professionals worked on the implementation of the WMP Brazil Communication and Community Engagement strategy. The actions presented in this paper refer to the work carried out between November 2016 and October 2018, in the municipalities of Rio de Janeiro and Niterói, in the state of Rio de Janeiro, Brazil.

This strategy was developed based on a Freire’s approach to community action ^30, 31^. The objective was not to convince the population about the benefits of the WMP *Wolbachia* method for reducing transmission of arboviruses, but, based on a critical understanding of the territory of operation, mapping and articulation with the social actors involved, to inform them about the intervention that would take place in the community, seek to foster critical discussions and dialogue on the role of the community and the various governmental and non-governmental actors in the control of arboviruses, the relationship between environment and health in the dissemination of these diseases and the role of science in health promotion.

In order to spread the information within the community, the communication and community engagement strategy was based on three areas of activity: public schools, basic health units and social leaders. The choice of these sectors was due to the networks created by them and, above all, the ability of these actors to understand the territory. In public schools, a partnership was made with the Municipal Education Secretariat of Rio de Janeiro and Niterói, and the teachers were trained to work with the WMP *Wolbachia* Method as an education action in science. After raising awareness about the method, the proposal was that each educator, in an autonomous way, would develop an action for the school community according to its reality, as proposed by Freire ^30, 32^. The understanding of the WMP Brazil team is that the control of arboviruses, the main theme of the actions, could not be discussed in a vertical manner, but rather from a problematisation of reality, since such diseases are, as mentioned above, directly linked to human interventions in nature.

To support the teaching actions, an e-book ^1^ focused on the prevention of arboviruses was prepared by the WMP Brazil engagement team. In this material, an environmental health approach was given to the theme. At the end of each chapter, some pedagogical activities were proposed, as a way to inspire and motivate teachers. Videos were also produced that could be used in the classroom or other scientific education spaces. These materials were made available on the WMP Brazil channel on YouTube and also, in the case of the city of Rio de Janeiro, on a digital access platform for teachers of municipal public schools.

In some schools in Rio de Janeiro and Niterói, in addition to classroom actions, a scientific experiment was organised using the Mosquito Release Container (MRC) ^2^. This equipment is used for the release of *Aedes aegypti* mosquito eggs with *Wolbachia* that has been adapted to be used as an instrument for science outreach. Students were invited to monitor the development of mosquitoes, and each teacher used this process in a pedagogical way that was more adjusted to their reality. Some addressed the various stages of the mosquito’s life, while others took advantage of the counting of eggs, larvae and pupae to work on mathematical content, among other possibilities. Overall, about three thousand teachers and directors of the municipal public schools in Rio de Janeiro and Niterói participated in the training and actions of WMP Brazil in the period from April 2017 to September 2019. In both municipalities, about 535 actions were carried out in educational spaces, mobilising around 103,600 people. Publicity materials were also produced, such as pamphlets, posters and children’s booklets, which were distributed to all schools and students in the municipal public school system in the intervention area.

For the health sector, a partnership was made with the Municipal Health Secretariat, in Rio de Janeiro and Niterói, for joint action in the territory. Training was carried out with the teams of the health units through techniques of using maps and reading of territory, dramatisation and expository presentations. Disclosure actions were also carried out within the health units, such as the waiting room, through stands and service groups.

In partnership with the municipality, it was defined that community health agents ^3^ would be responsible for bringing information and clarifying residents’ doubts about the *Wolbachia* Method in areas where health clinics operate. It is important to highlight that these professionals, as a rule, live in the neighbourhoods where they work or in nearby neighbourhoods, being, therefore, recognised leaders in the community itself. In addition, their critical reading of the territory helped to define other actions to be carried out, such as mapping social leaders, community communication channels and identifying hosts for traps that were used to monitor mosquitoes with *Wolbachia*. Health surveillance agents were also trained, as they were responsible for the release of mosquitoes and trap monitoring services in areas of socioenvironmental risk and vulnerability.

Communication materials were produced specifically for these professionals, and WhatsApp groups were created by the health unit. In these groups, information was released about the progress of the project in the area in which the unit operates, in addition to news and information about the *Wolbachia* Method. In addition to the virtual group, periodic visits were made by the WMP Brazil community engagement team to the units. In total, 1205 professionals from the health departments of Rio de Janeiro and Niterói were trained, and 62 WhatsApp groups were created.

The work with the social leaders took place after stakeholder mapping. The WMP Brazil community engagement team searched documents and websites about the territories in operation, as well as listening to health and education professionals who were partners of WMP. At each meeting or training session given by the team, it was asked who were the key leaders in the community we should engage. As soon as the indications started to repeat, it was understood that the main stakeholders had been identified. This mapping of stakeholders guided the performance of engagement and communication actions. Once the leaders were mapped, visits were scheduled to present and discuss the project and, when possible, a community event was organised. Communication materials were also produced to be disseminated on the partners’ social networks or via their WhatsApp channels. The mapping of stakeholders also sought to identify channels of communication through which it was possible to disseminate information on the method and involve the community to participate in field activities or even become a volunteer in monitoring mosquitoes.

In addition, press relations and social media actions were carried out, with the publication of content aimed at reaching certain age and social groups that were not present in the field actions that were carried out by the community engagement team. Profiles were created on social networks such as Facebook, Instagram and YouTube (@wmpbrasil). In addition, WhatsApp numbers, email addresses and phone numbers were released so that the population could contact them with questions and suggestions or complaints. These contacts were disclosed in all publicity materials and field actions.

The proposal of WMP Brazil was, once the political-social and economic dynamics of the territory were understood, to define actions of engagement or communication so that the different audiences were reached. In 30 months of fieldwork (from April 2017 to October 2019), approximately 1500 field activities were carried out, involving approximately 210 thousand people.

## Mosquito trap hosts and WMP Brazil Reference Groups

At the same time that the actions described above were carried out, with the objective of bringing information about WMP Brazil’s activities into the territories, promoting debates about the control of arboviruses and related environmental, sanitation and health issues, the WMP Brazil team was looking for volunteers who agreed to host a mosquito trap.

This action is fundamental for monitoring the invasion of *Ae. aegypti* with *Wolbachia* in the territories where the WMP operates and represents active participation of the community in the implementation of the WMP *Wolbachia* Method. Each volunteer received a mosquito trap, which was supposed to be connected to the electricity for 24 hours a day and, every week, a technician from WMP Brazil collected the mosquitoes. At any time, the host could request the removal of the equipment, if that person no longer wanted to participate.

All volunteers signed an informed consent form, in which the WMP *Wolbachia* Method, the operation of the trap, the reason for its installation, the routine for maintaining the equipment and collecting mosquitoes and how they would be reimbursed, to compensate the use of their electricity were all explained. Contact information of both the researcher responsible for WMP Brazil, as well as community engagement team contacts were also provided if the volunteer wanted to ask questions or request the removal of the equipment. The layout of the traps in the territory followed a grid of 250 by 250 meters, which meant that the community engagement team needed to find volunteers respecting this delimitation. To this end, a team of six technicians visited homes and commercial establishments to explain the *Wolbachia* Method and, if the citizen accepted, to install the traps. Overall, as of December 2019, 1411 active volunteers were participating in hosting a trap, in both municipalities (Rio de Janeiro and Niterói).

Another kind of direct community participation in the actions of WMP Brazil was through Community Reference Groups. It is an advisory committee created to enable the dialogue and monitoring of the project with local stakeholders. The composition of the group was based on the invitation of WMP Brazil to representatives from different sectors of the communities where the WMP operated. The invitation was made after the mapping of stakeholders, as described above.

Participation in the CRG was voluntary and meetings were held regularly (with meetings every 30–45 days). In these meetings the WMP team presented the progress of the project, the preliminary results of the invasion of mosquitoes with *Wolbachia* and discussed the plans and actions of communication and community engagement. Participants were free to debate, ask questions, criticise and make suggestions. This was a time for feedback and dialogue with community representatives. The discussions were recorded in the minutes and the groups remained mobilised for the duration of the WMP intervention in that particular territory.

The Rio de Janeiro CRG was composed of ten people, representing the following sectors: local health services, local public schools, university, residents’ association, non-governmental social action organisation, cyclists’ association, the local sub-prefecture and Municipal Guard. In Niterói, the CRG was also composed of ten people who represented the local health services, university, the local municipal administration, commercial association, recreational clubs, residents’ association and non-governmental social action organisation.

Each participant of the CRG signed an informed consent form and was enlisted in a WhatsApp group through which more brief and immediate communications were made, including the definition of the dates of the meetings.

## Reflections on WMP Brazil’s community engagement experience

We highlight three points of reflection based on the experience presented, which we consider to be essential for community engagement to fulfil its role, as described above in this article: 1) the territory is alive and needs to be constantly analysed, so that sociocultural particularities are identified and respected in the intervention area; 2) plural engagement actions are essential to reach different audiences, however, the basic value of all these actions must be the disposition for dialogue, or to put it another way, a willingness of the WMP teams and local actors to exchange information and experiences, understanding that none of the parties dominates all knowledge and that it is built from the lived experience and the relationship between the parties; 3) building capacity of the engagement team on the role of dialogue for the mobilisation of communities, with respect to their sociocultural bases, is essential when long-term and large-scale community engagement action is undertaken, such as that of WMP Brazil.

All community work aimed at engaging local actors requires a refined sociocultural reading ^12, 15, 33– 35^, with social leaders mapping, communication channels identification and means to favour community dialogue ^12, 34^. We identified that, as the territory is a live organism, the social compositions inherent to the space are fluid and are temporarily reorganised according to the experience; it is necessary that the engagement plan is able to be readjusted, maintaining the same values initially defined. In socio-environmentally vulnerable communities, and with a high level of violence, some actions needed to be adapted, such as, for instance, community events or visits to schools or health units. More than once, some scheduled activities needed to be canceled because police operations or armed disputes between rival drug dealers are taking place. As a result, some activities could not be carried out or were carried out at another time and / or location, which had an impact on the budget and schedule.

The communication and engagement strategies of WMP Brazil, for example, could not be watertight and should allow adjustments at the same time that they were implemented as actions in the community. Listening to local actors is active and can, at the same time as expanding some action, lead to the suppression of another that, for some reason given in that scenario, could no longer be carried out.

An example of our experience in Rio and Niterói, when doing field research on the main means of information for the population, as indicated by the Community Reference Group (GCR), television should be a vehicle for us to invest in the communication strategy. However, the value of television commercials in Brazil is very high and exceeded the budget, so a strategy of press relations was developed, so that there would always be journalistic coverage, especially of community media, following the suggestion of the GCR and observing the data indicated in the surveys, but within budget constraints. However, to build this relationship with the press, more time and specific work by the communication team was needed.

 Adjustments like this have an impact on the planning as a whole of a large-scale action such as that of WMP Brazil. There was a schedule and budget foreseen for the execution of community engagement that did not allow for major changes. In the example mentioned above, the use of advertisements on television could save us time and scale, with information reaching more people more quickly. The relationship with the press eliminated the cost, but increased the time of the action, affecting the schedule.

For those who are at the forefront of community mobilisation actions to combat arboviruses, it is necessary to reconcile the management aspects of the action without losing sight of the reasons and values that support community engagement; active listening, access to information and dialogue with the community. For that, the model proposed in Figure 1 seems to us to be relevant and effective when developing a community engagement plan. It is necessary to enable different actions, with different levels of involvement of local actors. This is because, not all actors are interested in participating actively, but it is necessary to allow these subjects access to information and, if they want to in another moment, to make active participation possible. At any time, you need to be available for dialogue, especially when it comes to the implementation of a method for controlling arboviruses resulting from scientific research. With dialogue, people think together, which assumes that the participant in the dialogue understands that his/her position is not the final one. It is a way of concentrating the energy of the differences that exist in the territory to create something new, and that requires a deep capacity to listen. Not just listening to the words of others, but letting yourself be influenced by them, causing a reflection.

In this relationship of mutual construction, it is necessary to emphasise that the dialogue will not replace the conflict, which is expected due to the diversity of opinions, interests and positions, by consensus, but, in the best of cases, make it constructive by focusing and clarifying the issues and critical points. Read ^36^ argues that dialogue can make the common good possible, since it is not natural among individuals, as each has different goals and preferences from each other. By making individual goals flexible, dialogue makes it possible to build a common good among the enormous diversity that is present in social life.

In order to build collective and dialogical processes in WMP Brazil’s engagement actions, it was decided to propose several activities with varying levels of participation and the opening of different listening channels. At the same time in which a digital communication strategy was defined via social media and the website, where citizens could be informed of all WMP actions, interaction channels were made possible, such as WhatsApp, telephone and emails; field actions, with an important physical presence for the exchange with the community; the training of professionals who worked in the territories, enabling the multiplication of scientific knowledge and the creation of reference groups, as spaces for listening and dialogue with the community.

The WMP Brazil Community Reference Groups, which functioned as local committees, were spaces for speaking and listening and had an influence on the actions, insofar as it made it possible for the WMP team, to understand what the potential of acting in the releasing area and what the limitations were. In addition to making it possible to listen to the concerns inherent in the process of releasing mosquitoes. The various actors present there expressed their concerns freely, especially at the beginning of the intervention, which was treated by the WMP team as an opportunity to understand the local cultural dynamics and readjust the actions and also the language, not with the aim of manipulating the community, but rather to provide citizens with better access to information so that they could take a position on the implementation of the method.

One of the concerns expressed by the group early in the work, for instance, was whether mosquitoes with Wolbachia could transmit other diseases. In order to respond to the group, contributions were made by scientists who clarified doubts until the topic was exhausted, and the group had no new questions. If, afterwards, any new question arose, it was done via WhatsApp group or answered at the next meeting of the group. In addition, the communication team understood that these doubts would need to be addressed in the engagement materials, such as pamphlets, social networks and presentations that were made by the community engagement team.

For this to happen, it was necessary to pay attention to the work of WMP Brazil professionals involved in communication and community engagement actions. As previously stated, the team was multidisciplinary, with several academic backgrounds and professional experiences, not all of which were initially aligned with this understanding of engagement as an act of listening and dialogue. Also, these people were exposed to different types of violence in Rio de Janeiro and Niteroi favelas during their field work. Thus, it was necessary to promote training actions and create, internally, further space for listening and dialogue.

To this end, rounds of conversation were held between team members, so that they could socialise the experiences and elaborate on the meanings arising from them. In this process, this dynamic was facilitated to provoke reflection, connecting with the values of listening, exchange and dialogue with the community. These internal sessions allowed the Community Engagament team to speak and listen, giving new meanings to their own performance and renewing the interests that kept them active in favour of the community.

## Considerations

As previously stated and explained in The Global Vector Control Response 2017–2030, of the World Health Organization, the rapid spread of arboviruses due to unplanned urban development, changes in land use, the impacts of climate change and the increase international mobility, indicates that it will not be possible to face these diseases except through cooperative action among the various social actors, including communities, academia and public management.

In view of this scenario, the work of community engagement for the control of arboviruses should not be understood only as a community intervention in public health, but also as an opportunity for local development. It is necessary, based on an integrated understanding of the territory, to understand that vector control actions or combating arboviruses can be a catalyst for the necessary socioenvironmental, cultural and public health changes.

The experience of WMP Brazil described in this document, indicates that in order to achieve such changes, it is essential to understand that community engagement goes beyond informing the population, but it must be a possibility of dialogue between the various actors present in the territories, so that there is cooperation for the common good, which, in this case, is the control of arboviruses.

## Data availability

### Underliyng data

All data underlying the results are available as part of the article and no additional source data are required.

## Footnotes

^1^ Available at wolbitonaescola.org.

^2^ An example is available at https://www.youtube.com/watch?v=0HgCI1kpl3g.

^3^ More than 1200 community health workers worked in the areas where the *Wolbachia* Method was being implemented. These public health professionals have the role of dialoguing with the population of the neighbourhoods, bringing them closer to the medical services offered. These workers also played a role in health education.
