# Supplementary material for: How to engage communities on a large scale? Lessons from World Mosquito Program in Rio de Janeiro, Brazil
Source: Gates Open Res. 2021 Feb 4;4:109. Originally published 2020 Jul 17. [Version 2] doi: 10.12688/gatesopenres.13153.2 (PMC7569240; doi:10.12688/gatesopenres.13153.2)
Supplement: Peer review history

# Peer review: Reviewer response for version 2

**DOI:** 10.21956/gatesopenres.14438.r30386

**Published:** 2021-02-17

**Authors:** Delphine Thizy

**Affiliations:** 1 Department of Life Sciences, Imperial College London, London, UK

The authors have done a good work addressing my comments as well as those from other reviewers. I find beneficial the addition of example, illustrating some of the challenges related to the specific context and more structural issues (such as the question of funding and deliverables flexibility). 

 I believe this paper will be an important contribution to the ongoing discussion in the field of innovative area-wide vector control interventions. It provides a good case study to the calls from WHO to plan for effective engagement and community mobilisation.

 It would be fascinating in the future to read about the comparison of WMP's engagement approach in different contexts to evaluate whether the model can be adapted to any culture and context based on the original stakeholder analysis or whether there are some challenges to that.

Does the article adequately reference differing views and opinions?

Yes

Are all factual statements correct, and are statements and arguments made adequately supported by citations?

Partly

Is the Open Letter written in accessible language?

Yes

Where applicable, are recommendations and next steps explained clearly for others to follow?

Partly

Is the rationale for the Open Letter provided in sufficient detail?

Yes

Reviewer Expertise:

vector control, stakeholder engagement, policy for innovation, communication, post-conflict contexts, social performance

I confirm that I have read this submission and believe that I have an appropriate level of expertise to confirm that it is of an acceptable scientific standard.

# Peer review: Reviewer response for version 1

**DOI:** 10.21956/gatesopenres.14347.r29552

**Published:** 2020-10-13

**Authors:** Trudie Lang

**Affiliations:** 1 Nuffield Department of Medicine, Centre for Tropical Medicine and Global Health, University of Oxford, Oxford, UK

Community engagement is a crucial and yet often over-looked, or undertaken as an almost token step, when conducting health research studies. It is also often considered to be an element related to the conduct of clinical trials or human intervention studies of some form.

 This article reports on the essential role of community engagement in arbovirus control and this is an excellently presented example of why community engagement is so fundamental and why it should be seen as a vital and core element of any planned research study.

 The authors describe their work in a balanced and considered manner and I congratulate them for writing up this vital element of their work because I think this serves their aim well of encouraging others to ensure this is also built into their planned studies.

 Community engagement should be thought about at the very start, when a protocol is first considered and then taken right through the project ending in making sure the community are informed of the results and how any recommendations are being put into practice so that they can benefit from the findings.

Does the article adequately reference differing views and opinions?

Yes

Are all factual statements correct, and are statements and arguments made adequately supported by citations?

Yes

Is the Open Letter written in accessible language?

Yes

Where applicable, are recommendations and next steps explained clearly for others to follow?

Yes

Is the rationale for the Open Letter provided in sufficient detail?

Yes

Reviewer Expertise:

NA

I confirm that I have read this submission and believe that I have an appropriate level of expertise to confirm that it is of an acceptable scientific standard.

# Peer review: Reviewer response for version 1

**DOI:** 10.21956/gatesopenres.14347.r29553

**Published:** 2020-09-30

**Authors:** Delphine Thizy

**Affiliations:** 1 Department of Life Sciences, Imperial College London, London, UK

This paper is an essential contribution of WMP to the on-going elaboration of a more explicit set of guidelines and best practices for the field of stakeholder engagement for innovative area-wide vector control. It provides a good overview of the overall engagement approach as well as their Public Acceptance Model and as such, can inform other practitioners or researchers. It is particularly interesting because WMP is one of the leaders in the field of innovative vector control strategies, having gone in the last years from very small scale interventions (such as documented in Kolopack et al. 2015) to now substantial scale engagement as described in this paper. 

 However, it would be interesting to explore a bit more some of the challenges of going to such scale. There are some brief allusions to some (e.g. issue of the flexibility of funding mechanisms to be responsive to requests, challenges in adapting to stakeholders' diverse interests by providing flexible engagement opportunities). Still, the paper would benefit from adding more details on the specific issues of changing scale. In particular, the tension or balance between the communication tools (social media, comms toolkits for schools, etc.) and the face-to-face engagement would deserve further development as this is a clear challenge of engaging at a large scale while keeping a model as the one presented by WMP. 

 The point about vector control response being a "catalyst for the necessary socioenvironmental, cultural and public health changes" is critical and bears the question of the potential expectations that this creates in the community and with stakeholders and how a programme like WMP positions itself concerning it. 

 A couple of factual/editing corrections suggested: 

- In the introduction: for the reference to the number of deaths and % of the population at risk, the WHO reference is for all vector-borne disease not only arboviruses.
- Sentence: "Identifying and managing these interests is also the role of community engagement, especially when the maximum expression of this is understood, still in reference to the model proposed by the Wellcome Trust ( Figure 1), as an action that enables dialogue between the parties." is not very clear (on particular what is meant by understanding the maximum expression of this).

Does the article adequately reference differing views and opinions?

Yes

Are all factual statements correct, and are statements and arguments made adequately supported by citations?

Partly

Is the Open Letter written in accessible language?

Yes

Where applicable, are recommendations and next steps explained clearly for others to follow?

Partly

Is the rationale for the Open Letter provided in sufficient detail?

Yes

Reviewer Expertise:

vector control, stakeholder engagement, policy for innovation, communication, post-conflict contexts, social performance

I confirm that I have read this submission and believe that I have an appropriate level of expertise to confirm that it is of an acceptable scientific standard, however I have significant reservations, as outlined above.

# Peer review: Reviewer response for version 1

**DOI:** 10.21956/gatesopenres.14347.r29111

**Published:** 2020-08-24

**Authors:** James V. Lavery

**Affiliations:** 1 Hubert Department of Global Health, Rollins School of Public Health, and Center for Ethics, Emory University, Atlanta, GA, USA

**General Observations**

 This paper makes a valuable contribution to the growing literature on community engagement associated with global health and global development programs in that it provides a detailed overview of the reasoning and processes of a large-scale engagement strategy for a major program. The World Mosquito Program (WMP) has established a strong reputation as a leader in arbovirus control, but also in engagement practices. As such, this description of its “Public Acceptance Model”(PAM)  is likely to be influential for other groups pursuing the deployment of Wolbachia mosquito technologies, and other related technologies that are introduced at population scales.

 In light of this potential for the WMP’s PAM model to shape engagement practices for the field, I offer some questions and a few cautionary comments intended to encourage a critical learning approach to the experiences with the model to date. The rich description offered in this paper provides an ideal opportunity to highlight several key aspects of the model that reflect ongoing challenges for community and stakeholder engagement, and avenues for improved reasoning and practice.

**General Comments**

1. The paper is usefully explicit in its description of many of the PAM model’s processes and activities. But the explanations and justifications for some of the reasoning underlying the model remain somewhat vague. For example, the authors claim “…that in order to achieve effective community engagement, it is necessary to map and analyse the local sociopolitical and economic context in order to develop a strategy adjusted to that territory and to enable mobilization.” (p. 3) They go on to say (p. 3) that this will “…allow each subject to be impacted and involved in a particular way.” But it is not clear either what the mapping and analysis of the sociopolitical and economic context entails, or how those activities inform the interactions with individual participants. 
2. A related issue arises in the PAM model’s core principle of “responsiveness”. The paper describes numerous engagements with various stakeholder groups, including municipalities and schools. The descriptions emphasize the relevant activities that the program undertook, but provide very few insights about what was *learned *through these activities. As a result it never became clear precisely what the program was being responsive *to*. Reading between the lines, it seems as though the program is being responsive to the preferences of stakeholders for how they would like to be engaged. If this is the case, it would have been helpful for that point to be made more explicitly. But if it is the case, it raises the question, above, regarding the purpose of a broader sociopolitical and economic analysis. The emphasis on this analysis suggests that “responsiveness” might have something to do with broader considerations in the context, rather than individual preferences for engagement. Some clarification on this point would be extremely helpful for readers and for the field.
3. At several points in the paper, the authors allude to, and in some cases make explicit references to, engagement as a participatory philosophy and as a vehicle for the education and development of communities, citing Massardier and Friere, for example. But these philosophies seem somewhat at odds with the PAM models explicit emphasis on community “acceptance”, which implies a persuasive role for engagement. It would be extremely helpful for readers to have a more explicit explanation of the meaning of “acceptance” in the PAM model and how it is informed by, and is congruent with, participatory development practices.
4. Picking up on the point about “acceptance”, in point #3, above, one of the chronic challenges for community engagement in the context of research programs that introduce new technologies is clarifying the researchers’ authority for asking individuals to “accept” a new technology, and what that “acceptance” means. In order for the WMP to conduct its work in Brazil it required a wide range of “formal” approvals by various levels of government. These government agencies have legislative mandates to act in the best interests of their citizens, and in this sense they are also stakeholders for engagement. The paper would be strengthened significantly—in my view—if the authors could provide more clarity on the terms of these approvals and the perceived value of the WMP program for the authorizing government agencies, and how they expect the technology to be utilized  beyond the introductory trials to facilitate the control of arbovirus disease transmission. This would provide a richer context for the interpretation of “acceptance” and help avoid any perception that the trials are solely of interest or value for the WMP.
5. The paper would be strengthened significantly by the addition of several specific examples or illustrations for key points. See Specific Comments, below. 

**Specific Comments** 

1. The paper refers, in several places, to the importance of dialogue. For example: “Assuming Massardier’s perspective that policymaking is to manage the collective actions of the actors, is to hold them together, no longer by authority, but by understanding the different rationality of each actor, we can understand that the dialogue fulfills a fundamental role in the management of a mosquito borne-disease policies.” (p. 4) . The quote calls into question the idea of authority—presumably of the policies that are enacted by formal authorities—in a way that is not easy to grasp. But, perhaps more importantly, it introduces the idea that dialogue plays a fundamental role in engagement. This would be an ideal opportunity for the authors to provide a brief explanation of that role in light of some of the general comments, above. What is dialogue? And what purpose does it serve in “public acceptance”?
2. On p. 4 the authors cite King et al. saying that “from an ethical point of view, the participation of the population builds legitimacy to scientific action…” and subsequently “(i)t is the role of community engagement, therefore, to respect and consider the different interests of the communities involved in combating arboviruses.” As an author on the King paper I think it would be helpful for readers to have a slightly more accurate account of the point that we make about legitimacy. On p. 3 of the King et al. paper we say that “community engagement embodies a democratic ideal in which legitimacy emerges from deliberative processes through which disagreement is acknowledged and addressed, lines of accountability are established between the stakeholder community and researchers, and stakeholders are empowered to ask directly for justification regarding the trial’s conduct and management.”
3. The first full paragraph on p. 6 discusses Freire’s “approach to community” action. The relevance of this paragraph would be strengthened significantly—in my view—by the addition of some information about the terms of the formal approvals for the trials, and their perceived value by the relevant government authorities. (See General Comment #4, above)
4. P. 8, “Reflections on WMP Brazil’s community engagement experience”: Three “points of reflection” for community engagement to fullfill it’s role: Point 1) “…so that sociocultural particularities are identified and respected in the intervention area.” Can the authors provide some examples of these “particularities” and the processes through which they were identified and respected? And who determined that the particularities were “respected”, and how. This would help readers understand the mechanics of the engagement strategy more fully. Point 2) “plural engagement actions are essential to reach different audiences, however, the basic value of all these actions must be the disposition for dialogue”. It would be helpful to clarify, here: (a) what is meant by the “disposition for dialogue”, and *who *is supposed to reflect this disposition, i.e., the program, the community, or both? And again, any further concreteness that could be offered in terms of examples or illustrations would be extremely helpful for readers.
5. P. 8, column 2, paragraph 2: “There was a schedule and budget foreseen for the execution of community engagement that did not allow for major changes.” What are the implications of this statement for the foundational claim in the PAM method that it is grounded in a commitment to “responsiveness”. Was the budget flawed? Is the commitment to responsiveness over-stated? Were there specific contextual factors that made certain types of responsiveness difficult or impossible? What were the implications?
6. P. 8, column 2, final paragraph: “…dialogue will not replace the conflict, which is expected due to the diversity of opinions, interests and positions, by consensus, but, in the best of cases, make it constructive by focusing and clarifying the issues and critical points.” It is unclear whether this statement alludes to the *specific *experiences of the WMP—in which case some specific examples or illustrations would be extremely helpful—or whether it is a more general point about the limitations of dialogue?
7. P. 9, column 1, first full paragraph, re. “Community Reference Groups”: “…which functioned as local committees, were spaces for speaking and listening and had an influence on the actions, insofar as it made it possible for the WMP team, to understand what the potential of acting in the releasing area and what the limitations were.” Given the central importance of “responsiveness” in the PAM model, it would be important here—in my view—for the authors to provide some specific examples or illustrations of the types of insights that arose in the context of the CRGs and the specific steps that were taken to be responsive to them, and, ideally, the assessment, by the CGRs, of the sufficiency and value of the responses.
8. P. 9, column 1, first full paragraph: “The various actors present there [i.e., in the CRGs] expressed their concerns freely, especially at the beginning of the intervention, which was treated by the WMP team as an opportunity to understand the local cultural dynamics and readjust the actions and also the language, not with the aim of manipulating the community, but rather to provide citizens with better access to information so that they could take a position on the implementation of the method.” This statement again offers an excellent opportunity for clarification and specification. What were some of the concerns? Who expressed them? Why did they express them, i.e., what interests were at stake for them? What were the learnings and improved understandings by the WMP? And how did these insights change or refine the WMP’s practices?
9. P. 9, column 2, “Considerations”, paragraph 2: “…the work of community engagement for the control of arboviruses should not be understood only as a community intervention in public health, but also as an opportunity for local development.” This point has a great deal of support in the community engagement literature. But there has been inadequate attention—in my view—of how the emphasis on community engagement as a vehicle for local development reconciles with the PAM method’s emphasis on “acceptance”, which is the subject of considerable debate, given its association, at times, with concepts such as “license to operate” and “engagement” practices from extractive industries. If the authors could provide even a brief description of how their commitment to local development informs the specific meaning of “acceptance” in the PAM model it would help readers to better understand and appreciate the model and its successes to date.

 
**Summary**

 This paper provides a valuable set of insights about one of the major challenges facing community engagement strategies, i.e., how can and should community engagement be designed and implementation in large-scale programs. In my comments, above, I have tried to identify several key points where additional clarity, and examples and illustrations, would be extremely valuable for readers and could improve the value of this paper’s contribution for the field.

Does the article adequately reference differing views and opinions?

Partly

Are all factual statements correct, and are statements and arguments made adequately supported by citations?

Partly

Is the Open Letter written in accessible language?

Yes

Where applicable, are recommendations and next steps explained clearly for others to follow?

Partly

Is the rationale for the Open Letter provided in sufficient detail?

Yes

Reviewer Expertise:

Global health ethics, stakeholder engagement, organizational learning, dialogue and deliberative methods, research ethics, qualitative case study methods

I confirm that I have read this submission and believe that I have an appropriate level of expertise to confirm that it is of an acceptable scientific standard, however I have significant reservations, as outlined above.

# Response

**Published:** 2020-12-30

**Authors:** Luciano Moreira

**Affiliations:** FIOCRUZ, Brazil

**The paper is usefully explicit in its description of many of the PAM model’s processes and activities. But the explanations and justifications for some of the reasoning underlying the model remain somewhat vague. For example, the authors claim “…that in order to achieve effective community engagement, it is necessary to map and analyse the local sociopolitical and economic context in order to develop a strategy adjusted to that territory and to enable mobilization.” (p. 3) They go on to say (p. 3) that this will “…allow each subject to be impacted and involved in a particular way.” But it is not clear either what the mapping and analysis of the sociopolitical and economic context entails, or how those activities inform the interactions with individual participants.**

*The socio-political and economic analysis allows us to understand the dynamics of the territory, the key actors present there, so that it is possible to outline a communication and engagement strategy, adjusted to the local reality, in which this manuscript sought to describe.*

**A related issue arises in the PAM model’s core principle of “responsiveness”. The paper describes numerous engagements with various stakeholder groups, including municipalities and schools. The descriptions emphasize the relevant activities that the program undertook, but provide very few insights about what was learned through these activities. As a result it never became clear precisely what the program was being responsive to. Reading between the lines, it seems as though the program is being responsive to the preferences of stakeholders for how they would like to be engaged. If this is the case, it would have been helpful for that point to be made more explicitly. But if it is the case, it raises the question, above, regarding the purpose of a broader sociopolitical and economic analysis. The emphasis on this analysis suggests that “responsiveness” might have something to do with broader considerations in the context, rather than individual preferences for engagement. Some clarification on this point would be extremely helpful for readers and for the field.**

*Responsiveness occurs in the ability to respond to the demands of different groups, either through the activities carried out or by accessing the available communication channels. By both means, the speeches of the community were heard, taken into consideration and answered directly.*

**At several points in the paper, the authors allude to, and in some cases make explicit references to, engagement as a participatory philosophy and as a vehicle for the education and development of communities, citing Massardier and Friere, for example. But these philosophies seem somewhat at odds with the PAM models explicit emphasis on community “acceptance”, which implies a persuasive role for engagement. It would be extremely helpful for readers to have a more explicit explanation of the meaning of “acceptance” in the PAM model and how it is informed by, and is congruent with, participatory development practices.**

*The term acceptance refers to an opinion survey that is carried out by an independent group before releases to investigate the understanding about the Wolbachia Method and whether the community accepts mosquito releases.*

*In the case of Brazil, the model was adjusted to the local reality and to the expertise of the team that would implement it. At the end of this experience, it is possible to reflect on the possibility of adjustments to the model, including changing its name, perhaps to Public Engagement Model.*

**Picking up on the point about “acceptance”, in point #3, above, one of the chronic challenges for community engagement in the context of research programs that introduce new technologies is clarifying the researchers’ authority for asking individuals to “accept” a new technology, and what that “acceptance” means. In order for the WMP to conduct its work in Brazil it required a wide range of “formal” approvals by various levels of government. These government agencies have legislative mandates to act in the best interests of their citizens, and in this sense they are also stakeholders for engagement. The paper would be strengthened significantly—in my view—if the authors could provide more clarity on the terms of these approvals and the perceived value of the WMP program for the authorizing government agencies, and how they expect the technology to be utilized  beyond the introductory trials to facilitate the control of arbovirus disease transmission. This would provide a richer context for the interpretation of “acceptance” and help avoid any perception that the trials are solely of interest or value for the WMP.**

*To operate in Brazil, the WMP obtained authorization from regular bodies and also from the National Research Ethics Commission. This information was added to the article.*

**The paper refers, in several places, to the importance of dialogue. For example: “Assuming Massardier’s perspective that policymaking is to manage the collective actions of the actors, is to hold them together, no longer by authority, but by understanding the different rationality of each actor, we can understand that the dialogue fulfills a fundamental role in the management of a mosquito borne-disease policies.” (p. 4) . The quote calls into question the idea of authority—presumably of the policies that are enacted by formal authorities—in a way that is not easy to grasp. But, perhaps more importantly, it introduces the idea that dialogue plays a fundamental role in engagement. This would be an ideal opportunity for the authors to provide a brief explanation of that role in light of some of the general comments, above. What is dialogue? And what purpose does it serve in “public acceptance”?**

*The notion of dialogue is quoted in the excerpt: By dialogue, we understand that it is a way to concentrate the energy of the differences present in the territory to build something new that brings benefits for collectivity. In the dialogue, there are no sides, not either someone who has an exclusive knowledge.*

*Regarding the term public acceptance, as already mentioned in previous comments, it refers to the data resulting from the pre-release survey.*

*This Brazilian experience may assist in revising the model that was created when the method was not yet implemented on a large scale.*

**On p. 4 the authors cite King et al. saying that “from an ethical point of view, the participation of the population builds legitimacy to scientific action…” and subsequently “(i)t is the role of community engagement, therefore, to respect and consider the different interests of the communities involved in combating arboviruses.” As an author on the King paper I think it would be helpful for readers to have a slightly more accurate account of the point that we make about legitimacy. On p. 3 of the King et al. paper we say that “community engagement embodies a democratic ideal in which legitimacy emerges from deliberative processes through which disagreement is acknowledged and addressed, lines of accountability are established between the stakeholder community and researchers, and stakeholders are empowered to ask directly for justification regarding the trial’s conduct and management.”**

*The authors appreciate the recommendation. The adjustment was made in the text.*

**The first full paragraph on p. 6 discusses Freire’s “approach to community” action. The relevance of this paragraph would be strengthened significantly—in my view—by the addition of some information about the terms of the formal approvals for the trials, and their perceived value by the relevant government authorities. (See General Comment #4, above).**

*As informed, the information was inserted in the text.*

**P. 8, “Reflections on WMP Brazil’s community engagement experience”: Three “points of reflection” for community engagement to fullfill it’s role: Point 1) “…so that sociocultural particularities are identified and respected in the intervention area.” Can the authors provide some examples of these “particularities” and the processes through which they were identified and respected? And who determined that the particularities were “respected”, and how. This would help readers understand the mechanics of the engagement strategy more fully. Point 2) “plural engagement actions are essential to reach different audiences, however, the basic value of all these actions must be the disposition for dialogue”. It would be helpful to clarify, here: (a) what is meant by the “disposition for dialogue”, and who is supposed to reflect this disposition, i.e., the program, the community, or both? And again, any further concreteness that could be offered in terms of examples or illustrations would be extremely helpful for readers.**

*The requested information was included in the text.*

**P. 8, column 2, paragraph 2: “There was a schedule and budget foreseen for the execution of community engagement that did not allow for major changes.” What are the implications of this statement for the foundational claim in the PAM method that it is grounded in a commitment to “responsiveness”. Was the budget flawed? Is the commitment to responsiveness over-stated? Were there specific contextual factors that made certain types of responsiveness difficult or impossible? What were the implications?**

*In some cases, territorial dynamics and the relationship with the community indicated actions that would not be possible due to a budget constraint. We cite here an example that was also included in the text: when doing field survey on the main means of information for the population, as indicated by the Community Reference Group, television should be a vehicle for us to invest in the communication strategy. However, the value of television commercials in Brazil is very high and exceeded the budget, so a strategy of press relations was developed, so that there would always be television news coverage, following the suggestion of the GCR, but within budgetary limitations.*

**P. 8, column 2, final paragraph: “…dialogue will not replace the conflict, which is expected due to the diversity of opinions, interests and positions, by consensus, but, in the best of cases, make it constructive by focusing and clarifying the issues and critical points.” It is unclear whether this statement alludes to the specific experiences of the WMP—in which case some specific examples or illustrations would be extremely helpful—or whether it is a more general point about the limitations of dialogue?**

*The reviewer is correct, as it is a reflection on dialogue.*

**P. 9, column 1, first full paragraph, re. “Community Reference Groups”: “…which functioned as local committees, were spaces for speaking and listening and had an influence on the actions, insofar as it made it possible for the WMP team, to understand what the potential of acting in the releasing area and what the limitations were.” Given the central importance of “responsiveness” in the PAM model, it would be important here—in my view—for the authors to provide some specific examples or illustrations of the types of insights that arose in the context of the CRGs and the specific steps that were taken to be responsive to them, and, ideally, the assessment, by the CGRs, of the sufficiency and value of the responses.**

*Added to the text.*

**P. 9, column 1, first full paragraph: “The various actors present there [i.e., in the CRGs] expressed their concerns freely, especially at the beginning of the intervention, which was treated by the WMP team as an opportunity to understand the local cultural dynamics and readjust the actions and also the language, not with the aim of manipulating the community, but rather to provide citizens with better access to information so that they could take a position on the implementation of the method.” This statement again offers an excellent opportunity for clarification and specification. What were some of the concerns? Who expressed them? Why did they express them, i.e., what interests were at stake for them? What were the learnings and improved understandings by the WMP? And how did these insights change or refine the WMP’s practices?**

*Added to the text.*

**P. 9, column 2, “Considerations”, paragraph 2: “…the work of community engagement for the control of arboviruses should not be understood only as a community intervention in public health, but also as an opportunity for local development.” This point has a great deal of support in the community engagement literature. But there has been inadequate attention—in my view—of how the emphasis on community engagement as a vehicle for local development reconciles with the PAM method’s emphasis on “acceptance”, which is the subject of considerable debate, given its association, at times, with concepts such as “license to operate” and “engagement” practices from extractive industries. If the authors could provide even a brief description of how their commitment to local development informs the specific meaning of “acceptance” in the PAM model it would help readers to better understand and appreciate the model and its successes to date.**

*We have already explained above about the term acceptance.*

**Thizy**

****

**It would be interesting to explore a bit more some of the challenges of going to such scale. There are some brief allusions to some (e.g. issue of the flexibility of funding mechanisms to be responsive to requests, challenges in adapting to stakeholders' diverse interests by providing flexible engagement opportunities).**

*Added to the text.*

**The paper would benefit from adding more details on the specific issues of changing scale. In particular, the tension or balance between the communication tools (social media, comms toolkits for schools, etc.) and the face-to-face engagement would deserve further development as this is a clear challenge of engaging at a large scale while keeping a model as the one presented by WMP.**

*Added to the text.*

**The point about vector control response being a "catalyst for the necessary socioenvironmental, cultural and public health changes" is critical and bears the question of the potential expectations that this creates in the community and with stakeholders and how a programme like WMP positions itself concerning it.**

*Throughout the engagement and communication work, the population is constantly informed that the Wolbachia Method is complementary and that it must be implemented in integration, along with other existing methods such as insecticides, mechanical control, among others.*

**In the introduction: for the reference to the number of deaths and % of the population at risk, the WHO reference is for all vector-borne disease not only arboviruses.**

*The reviewer is correct, many thanks. The correction was made on the text.*

**"Identifying and managing these interests is also the role of community engagement, especially when the maximum expression of this is understood, still in reference to the model proposed by the Wellcome Trust ( Figure 1), as an action that enables dialogue between the parties." is not very clear (on particular what is meant by understanding the maximum expression of this).**

*The maximum expression of community engagement, based on the Welcome Trust model presented, are the actions that allow dialogue between the parties.*
